# Hourly oral sodium chloride for the rapid and predictable treatment of hyponatremia

**DOI:** 10.5414/CN108014

**Published:** 2013-07-02

**Authors:** Eric Kerns, Shweta Patel, David M. Cohen

**Affiliations:** 1 1Division of Nephrology & Hypertension, Department of Medicine, Oregon Health & Science University, Portland, OR, USA, and; 2 Portland V.A. Medical Center, Portland, OR, USA

**Keywords:** osmoregulation, arginine vasopressin, hyponatremia, sodium chloride

## Abstract

Hypertonic NaCl is first-line therapy for acute, severe and symptomatic hyponatremia; however, its use is often restricted to the intensive care unit (ICU). A 35-year-old female inpatient with an optic chiasm glioma and ventriculoperitoneal shunt for hydrocephalus developed acute hyponatremia (sodium 122 mEq/L) perhaps coinciding with haloperidol treatment. The sum of her urinary sodium and potassium concentrations was markedly hypertonic vis-à-vis plasma; it was inferred that serum sodium concentration would continue to fall even in the complete absence of fluid intake. Intravenous (IV) 3% NaCl was recommended; however, a city-wide public health emergency precluded her transfer to the ICU. She was treated with hourly oral NaCl tablets in a dose calculated to deliver the equivalent of 0.5 mL/kg/h of 3% NaCl with an objective of increasing the serum sodium concentration by 6 mEq/L. She experienced a graded and predictable increase in serum sodium concentration. A slight overshoot to 129 mEq/L was rapidly corrected with 0.25 l of D_5_W, and she stabilized at 127 mEq/L. We conclude that hourly oral NaCl, in conjunction with careful monitoring of the serum sodium concentration, may provide an attractive alternative to IV 3% NaCl for selected patients with severe hyponatremia.

## Introduction 

Hyponatremia is a common electrolyte abnormality affecting 15 – 30% of hospitalized patients [1, 2]. Severe hyponatremia can be lethal; however, even modest changes in serum sodium concentration cause reversible defects in cognition and coordination [3] which can increase the risk of traumatic fracture [4, 5]. 

Since its first clinical application in 1938 [6], IV hypertonic (e.g., 3%) NaCl solution has been the primary therapy for severe, acute, and symptomatic hyponatremia [7, 8, 9]. Recent refinements to the use of hypertonic NaCl have focused on controlling and moderating the rate of increase in the serum sodium concentration [8]. Administration of hypertonic NaCl generally requires an intensive care unit setting [10]; an alternative approach obviating these limitations could prove attractive. 

We report our results with hourly administration of oral sodium chloride tablets for the partial correction of severe acute hyponatremia in a 35-year-old woman, and propose that this approach may be appropriate for first-line therapy in selected patients with severe hyponatremia. 

## Case report 

A 35-year-old woman presented to the emergency room with worsening of chronic abdominal pain. She had also developed progressive lower extremity edema over the prior several months and was treated with diuretics. She had been diagnosed with a glioma of the optic chiasm ~ 2 decades prior, for which she received chemotherapy and radiation. Following treatment, she developed anterior hypopituitarism, and required ventriculoperitoneal shunt for hydrocephalus. Medications (all chronic) included methadone, acetaminophen-hydrocodone, cyclobenzaprine, sumatriptan, ondansetron, divalproex sodium, gabapentin, low-dose furosemide, estrogen replacement, somatotropin, potassium chloride and vitamin D. 

On examination in the emergency room, she was afebrile with a blood pressure of 96/69 mmHg, pulse of 63, and weight of 40 kg. She was cachectic and non-toxic-appearing. Mucosae were moist. Jugular venous pulsations were not observed. Cardiopulmonary examination was unremarkable. The abdomen was moderately distended and firm with a fluid wave. There was 1+ peripheral edema. A limited neurologic examination was without deficit. 

Initial labs (Table 1) were notable for a serum sodium of 132 mEq/L (138 mEq/L 3 months prior), and a serum creatinine of 1.2 mg/dL (prior baseline 0.7 – 0.8 mg/dL). Contrast computed tomography showed new large-volume ascites. Magnetic resonance imaging of the brain showed a glioma invading the optic chiasm and the optic tract, predominantly on the left, unchanged from prior examination. 

**Figure 1.**
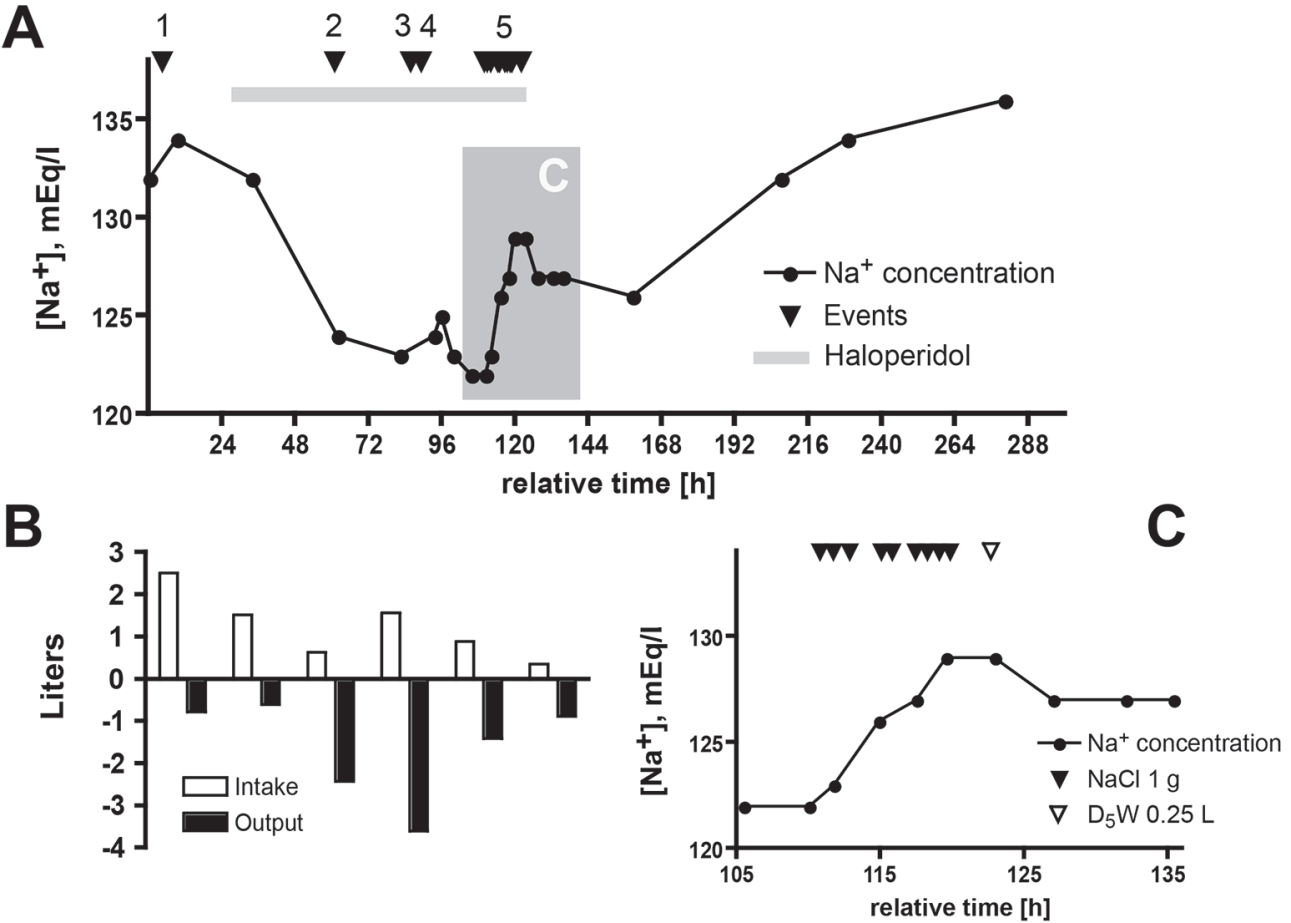
Data reflecting the clinical course. A: Trajectory of serum sodium concentration (mEq/L) as a function of time (in hours). Events (marked on timeline as arrowhead) are as follows: 1 – intravenous administration of 1 l normal saline; 2 – large-volume paracentesis of 3.2 l ascitic fluid; 3 – administration of 0.5 l of normal saline; 4 – imposition of 1.5 l/d fluid restriction; and 5 – treatment with oral NaCl tablets. The interval during which haloperidol was administered (total of 7 mg divided in 14 oral and parenteral doses) is marked with a horizontal gray bar. The shaded area (marked “C”) is expanded in Panel C. The final four [Na^+^] determinations were obtained as an outpatient. B: Recorded fluid intake and urinary output (in l) in 24-hour intervals corresponding approximately to the x-axis timeline in Panel A; data for the 6^th^ day are partial (incomplete), and data were not recorded beyond Day 6. The 24-hour intervals in B deviate by 4 hours from the interval in Panel A (time: 21:00 – 21:00 in A; 01:00 – 01:00 in B). Although not evident from the daily totals in B, much of the copious urine output on the 3^rd^ and 4^th^ hospital days (i.e., between hours 48 – 96) spontaneously occurred during the 8-hour overnight interval centered on Hour 72 in Panel A and totaled 2.6 l. C: Detailed trajectory of serum sodium concentration (representing shaded interval in Panel A) in response to hourly administration of NaCl (1 g tablets; filled arrowhead for each dose). Although prescribed hourly, the timing of administration was variable; depicted data reflect time of actual NaCl administration. At a serum [Na^+^] of 129 mEq/L, D_5_W (0.25 l) was administered intravenously (open arrowhead) with a resultant decrease in serum [Na^+^] to 127 mEq/L.

In addition to anti-emetics and narcotic analgesics, she received 1 liter of IV isotonic saline on the first hospital day. Haloperidol was begun for anxiety and in the ensuing 4 days, the patient received a total of 7 mg. By the second hospital day, renal function had returned to baseline. Serum sodium concentration decreased to 124 mEq/L on the 3^rd^ day (Figure 1A). On transthoracic echocardiogram, there was normal left ventricular size and function. The inferior vena cava was normal in caliber with appropriate inspiratory collapse. Paracentesis was performed and she received additional isotonic saline. Urine output increased during the night of the third hospital day, to 2.6 l total for the 8-hour interval between 20:00 and 04:00 of the 4^th^ day. On the 4^th^ day, serum sodium concentration was 123 mEq/L and nephrology consultation was obtained. 

At the time of consultation, there were no postural symptoms with ambulation. The blood pressure was 125/87, and the pulse was 66; there was no fever. Mucosae were moist and the jugular venous pressure could not be estimated. Cardiopulmonary examination was unremarkable. A small amount of ascites was present, there was no peripheral edema, and her sensorium was clear. Pertinent laboratory data are shown in Table 1. She was given a presumptive diagnosis of the syndrome of inappropriate antidiuresis based upon presumed intravascular euvolemia, multiple potentially offending medications, and the absence of urinary sodium avidity. Recommendations were to discontinue haloperidol, reduce divalproex and restrict fluids; however, in light of the substantial urine output (Figure 1B) and her urinary (Na^+^ + K^+^) far exceeding her serum (Na^+^ + K^+^), it was inferred that hyponatremia would worsen with no fluid intake. Intravenous infusion of 3% NaCl solution was recommended; however, a city-wide public health emergency (a local mass shooting) precluded ICU transfer. The sodium concentration transiently increased slightly, then fell to 122 mEq/L. The duration of the public health emergency was indeterminate and, after 24 hours, the patient had still not been accepted to the ICU. Her sensorium remained clear. With a concern for possible increase in intracranial pressure, a decision was made to semi-urgently increase serum sodium concentration on the regular hospital ward with hourly NaCl tablets. An oral dosing regimen was designed to mimic a 3% NaCl infusion rate of 0.5 mL/kg/h. Her mass of 40 kg would necessitate a 20 mL/h infusion of 3% (i.e., 3 g/dL) NaCl, or 0.6 g/h of NaCl. For 1-g tablets of NaCl, this equates to 0.6 tablets per hour; this was rounded up to 1 tablet per hour in light of the urinary cation loss. (Of note, where she to have become acutely symptomatic, a more rapid rate of 3% NaCl infusion (e.g., 1 – 2 mL/kg/h) would have been targeted or used to inform the oral dosing regimen). The treatment schedule and resultant laboratory data are shown in Figure 1C. The goal was an increment in serum sodium concentration of ~ 6 mEq/L. The patient readily adhered to this regimen, and experienced a near-linear increase in serum sodium concentration. Eight hours into treatment, the serum sodium concentration was 129 mEq/L; NaCl supplementation was stopped and she received a 250 mL IV bolus of 5% dextrose in water (D_5_W) with rapid stabilization of the serum sodium concentration at 127 mEq/L (Figure 1C). She was discharged on 2 gm NaCl supplementation daily. The day following discharge, her serum sodium was 126 mEq/L, and 2 days later, it had risen to 132 mEq/L, at which time NaCl supplementation was discontinued. 

## Discussion 

To our knowledge, there are no prior reports of the use of hourly oral sodium chloride tablets for the rapid and predictable treatment of severe hyponatremia. Oral sodium chloride supplementation is commonly used after acute correction to help sustain a response to 3% NaCl solution. Alternatively, oral sodium chloride may comprise an element of a chronic outpatient maintenance regimen for the treatment of euvolemic hyponatremia [7]. Woo et al. [11] incorporated sodium chloride tablets in a prophylactic regimen for neurosurgical patients. Our inability to secure intensive care unit monitoring – owing to an unfolding city-wide public health emergency – was the basis for our formulating and implementing this strategy. We anticipate that it could prove useful for other carefully selected cases of severe hyponatremia. 

A limitation of this approach is its requirement for active patient participation and adherence. Many clinical scenarios necessitating an urgent increase in the serum sodium concentration are associated with an altered sensorium; reliable adherence to an oral regimen cannot be assumed. In addition, although ICU-level care was not required to administer this regimen, intensive monitoring of the serum sodium concentration response to intervention was essential. Therefore, where nursing and/or physician manpower resources are limited, this approach may not prove advantageous. Whereas some have argued that hypertonic NaCl therapy should be reserved for the ICU [10], others routinely administer IV 3% NaCl outside of the ICU setting (e.g., [12]); the oral loading approach described here may offer fewer advantages in the latter environments. 

It could be argued that urgently increasing the serum sodium concentration was not essential in this setting. Although the patient was not overtly symptomatic, the magnitude of the acute fall in serum sodium concentration was concerning and, based upon her extensive CNS pathology, we considered her particularly sensitive to the adverse effects of even mildly increased intracranial pressure. Most notably, her urinary electrolyte concentration (Na^+^ + K^+^) was hypertonic with respect to her plasma such that a progressive fall in serum sodium concentration was anticipated even in the absence of additional fluid intake. The importance of the sum of the urinary sodium and potassium concentration vis-à-vis maintenance of the serum sodium concentration formed the basis for the Edelman equation [13], and has received renewed emphasis (e.g., [9, 14, 15]). Furthermore, the distinction between the presence vs. absence of neurologic symptoms in hyponatremia is somewhat artificial [12]; most hyponatremic patients have at least subtle symptoms (e.g., [3]). For these reasons, we felt that urgent partial correction of her serum sodium concentration was indicated. 

The rate of correction remained relatively constant (Figure 1A, C). A slight overshoot occurred (1 – 2 mEq/L) and – given the negative electrolyte-free water clearance – was rapidly corrected with a modest (0.25 l) infusion of free water (D_5_W). Re-lowering affords protection from adverse sequelae [16, 17, 18]. A prudent target for partial correction – in both acute and chronic hyponatremia – is an increment of 6 mEq/L within the first 24 hours. This is sufficient to prevent impending central nervous system decompensation in the acute setting [19], and to minimize the risk of myelinolysis in chronic hyponatremia [20]. 

A number of chronic medications could have contributed to the development of hyponatremia in this case, including narcotics [21] and valproic acid [22, 23, 24, 25]. Although most diuretic-induced hyponatremia is caused by thiazide diuretics [26], some cases are attributable to loop diuretics [27] such as furosemide in the present case. 

The acute administration of haloperidol was potentially instrumental [28]. Haloperidol was prescribed as an anxiolytic for this benzodiazepine-allergic patient; its discontinuation was recommended by the consulting nephrologist but implementation was delayed. Therefore, the effective correction of the hyponatremia by supplemental oral NaCl was not confounded by cessation of haloperidol therapy. The sudden increase in urinary output – occurring principally during the night between the 3^rd^ and 4^th^ hospital days – would be unexpected were this to represent purely haloperidol-induced SIAD. We do not have a satisfactory explanation for the transient polyuria; it did not appear to be a water diuresis as the effect upon the serum sodium concentration was minimal at best (Figure 1A). Of note, the mild acute kidney injury had resolved by the 2^nd^ hospital day. It seems likely that unrecorded oral intake of hypotonic fluid coincided with the development of hyponatremia during the 2^nd^ hospital day. 

A central basis for the hyponatremia was also considered. Gliomas arising from the optic chiasm have been associated with hypernatremia from central diabetes insipidus or osmoreceptor dysfunction [29]; hyponatremia/SIAD has been reported following surgery [30] and de novo in a case with features similar to the present one [31]. Abnormal adrenocortical and thyroid function can accompany pituitary failure and can give rise to an SIAD-like picture (reviewed in: [7]). This mechanism was not felt to be operative in the development of the acute inpatient hyponatremia, and her pituitary function had been closely monitored. Laboratory studies ~ 3 months prior to this admission were consistent with normal thyroid and adrenal function, and normal plasma levels of TSH and ACTH, respectively (data not shown). 

Although gastrointestinal symptoms comprised the admitting complaint, and although ascites was present, there were no clinical or laboratory findings to suggest that chronic liver disease was confounding the water balance picture (data not shown). Ascites was tentatively attributed to the presence of the ventriculoperitoneal shunt (e.g., [32]). Furthermore, were cirrhosis the basis for the water avidity in the present case, an extremely low urinary sodium concentration would be expected. 

The potential benefits of this hourly oral NaCl regimen include reduced cost, reduced reliance upon ICU resources, reduced need for central venous access, and a reduced number of patient care “hand-offs” obligated by team/unit transfer. In addition, this therapy can be started immediately upon recognition of hyponatremia – particularly in facilities such as our own where institutional policy precludes administration of intravenous hypertonic NaCl outside of an ICU setting. Delays are common in implementing NaCl therapy for hyponatremia [12]. Ward stocking with NaCl tablets might reduce or avoid the potential for errors in medication administration that has resulted in restricted distribution and stocking of 3% NaCl solution. We conclude that hourly oral NaCl supplementation – in conjunction with careful monitoring of the serum sodium concentration – may provide safe and effective therapy in selected patients with severe hyponatremia, and that this approach affords potential advantages over existing regimens. 

## Acknowledgments 

This work is supported by grants from the National Institutes of Health, the Department of Veterans Affairs, and the American Heart Association. The authors have no conflict of interest related to the contents of this manuscript. 

**Table 1:** Laboratory data obtained at admission and at time of nephrology consultation.

Determination	Value: admission	Value: time of consultation
Serum Na^+^ concentration	132 mEq/L	122 mEq/L
Serum K^+^ concentration	4.4 mEq/L	4.3 mEq/L
Serum creatinine	1.2 mg/dL	0.7 mg/dL
Serum osmolality		251 mOsmol/kg H_2_O
Urine osmolality		410 mOsmol/kg H_2_O
Urine Na^+^ concentration		138 mEq/L
Urine K^+^ concentration		21 mEq/L
